# Feasibility and potential significance of rapid *in vitro* qualitative phenotypic antimicrobial susceptibility testing of gram-negative bacilli with the ProMax system

**DOI:** 10.1371/journal.pone.0249203

**Published:** 2021-03-26

**Authors:** Jade Chen, Michael Tomasek, Amorina Cruz, Matthew L. Faron, Dakai Liu, William H. Rodgers, Vincent Gau

**Affiliations:** 1 GeneFluidics, Los Angeles, California, United States of America; 2 The Medical College of Wisconsin, Milwaukee, Wisconsin, United States of America; 3 Department of Pathology and Clinical Laboratories, NewYork-Presbyterian Queens, Flushing, New York, United States of America; 4 Department of Pathology and Laboratory Medicine, Weill Cornell Medical College, New York, New York, United States of America; Manipal College of Medical Sciences, NEPAL

## Abstract

The emergence and evolution of antibiotic resistance has been accelerated due to the widespread use of antibiotics and a lack of timely diagnostic tests that guide therapeutic treatment with adequate sensitivity, specificity, and antimicrobial susceptibility testing (AST) accuracy. Automated AST instruments are extensively used in clinical microbiology labs and provide a streamlined workflow, simplifying susceptibility testing for pathogenic bacteria isolated from clinical samples. Although currently used commercial systems such as the Vitek2 and BD Phoenix can deliver results in substantially less time than conventional methods, their dependence on traditional AST inoculum concentrations and optical detection limit their speed somewhat. Herein, we describe the GeneFluidics ProMax lab automation system intended for a rapid 3.5-hour molecular AST from clinical isolates. The detection method described utilizes a higher starting inoculum concentration and automated molecular quantification of species-specific 16S rRNA through the use of an electrochemical sensor to assess microbiological responses to antibiotic exposure. A panel of clinical isolates consisting of species of gram-negative rods from the CDC AR bank and two hospitals, New York-Presbyterian Queens and Medical College of Wisconsin, were evaluated against ciprofloxacin, gentamicin, and meropenem in a series of reproducibility and clinical studies. The categorical agreement and reproducibility for *Citrobacter freundii*, *Enterobacter cloacae*, *Escherichia coli*, *Klebsiella aerogenes*, *Klebsiella oxytoca*, *Klebsiella pneumoniae*, and *Pseudomonas aeruginosa* were 100% and 100% for ciprofloxacin, 98.7% and 100% for gentamicin and 98.5% and 98.5% for meropenem, respectively.

## Introduction

The widespread—and often inappropriate—use of antimicrobial agents partially due to the lack of rapid, evidence-based identification (ID)/antimicrobial susceptibility testing (AST) results is an important cause of the emergence and acceleration of drug resistance, with epidemiological studies demonstrating a direct relationship between antibiotic consumption and the emergence and dissemination of resistant bacteria strains [1,2]. Subinhibitory antibiotic concentrations can promote the development of antibiotic resistance by selecting for genetic alterations, such as changes in gene expression, horizontal gene transfer, and mutagenesis [3]. Antibiotics also remove drug-sensitive competitors, leaving resistant bacteria behind to reproduce as a result of natural selection [4]. Several studies have proven that rapid detection and treatment can reduce patient mortality by 50% or more and reduce hospital and intensive care unit length of stay by 3 to 7 days [5–7].

A recent review from the European Committee on Antimicrobial Susceptibility Testing concluded that there is currently insufficient evidence to support the use of resistance gene detection or whole genome sequencing (WGS) inferred AST for most bacterial species to guide clinical decision making. Some known limitations of these methods are poor correlation to culture-based AST and incomplete knowledge of the genetic basis of resistance [8–10]. Additionally, antibiotic resistance mechanisms of most pathogens are diverse and will continue to evolve. WGS would serve as an effective screening tool for the tracking of known antimicrobial resistance mechanisms. Therefore, rather than replacing phenotypic susceptibility testing, genotypic testing acts as a supplementary tool which could help to exclude the use of some specific types of antibiotics by detection of corresponding drug resistance genes [11].

In an ongoing effort to develop a rapid streamlined ID/AST system, we first investigated the AST portion with the use of our current automated rapid AST platform, the ProMax, intended to measure accelerated microbial responses to antibiotic conditions in a 3.5-hour AST. This system utilizes an electrochemical-based sandwich hybridization test used to determine qualitative antimicrobial susceptibility from direct suspensions of clinical isolates grown on solid media. Through validation studies, we determined the assay parameters, specifically antibiotic exposure time and inoculum concentration, necessary to achieve ≥ 95% categorical agreement with disk diffusion using three challenging conditions: (1) bacteria with longer doubling times, (2) time-dependent antibiotics, and (3) bacteria with minimum inhibitory concentrations (MIC) on or near susceptible and resistant breakpoints. These studies demonstrate our optimization efforts and initial evaluation of the feasibility of the current ProMax AST assay to be used in a future 10 to 12-hour fully automated streamlined ID/AST direct from clinical specimens with the aim of significantly reducing the time to results (S1 Fig), ultimately leading to more appropriate antibiotic use.

## Materials and methods

In accordance with federal regulations 45CFR 46.101 (b) and 21CFR56.104, the NYP/Queens IRB has reviewed and determined that the referenced protocol qualifies for exempt status under category #4.

### Study design

Given the fixed 2-hour AST incubation time used by ProMax, we designed two studies based on the guidelines in the FDA Class II Special Controls Guidance Document: AST Systems with Short Incubation Times (<16 hours) [12]: (1) reproducibility study and (2) clinical validation. Results were assessed by categorical agreement with CLSI disk diffusion. Reproducibility studies were first completed with ATCC, CDC, and International Health Management Associates (IHMA) strains to finalize assay design, which was then tested against New York-Presbyterian Queens (NYPQ) Hospital and Medical College of Wisconsin (MCW) strains as clinical validation. Although our system reported categorical susceptibility and was compared to disk diffusion results, clinical strains in each study were selected to cover a range of MIC values for each antibiotic. We wanted to challenge the ability of our system to consistently distinguish between susceptible and resistant strains on or near (± 2-fold dilution) the CLSI breakpoints, since this range of MIC values may present more difficulty in susceptibility reporting and lead to more major and very major discrepancies compared to strains that are highly susceptible or resistant. We aimed to test a variety of clinically relevant species as shown in S1 Table.

#### Reproducibility studies

We performed three separate reproducibility studies to assess the robustness and stability of assay results across different days. Day-to-day reproducibility was calculated within each study, not across studies. In each study, two operators tested from a panel of strains comprised of 39 CDC AR Bank isolates, 3 ATCC isolates (25922, BAA-2146, 27853), and 1 IHMA isolate once a day for 2–3 days. Specifically, studies 1, 2, and 3 had a panel of 28, 39, and 39 strains tested 2–3 times for a total of 62, 116, and 168 results, respectively, per antibiotic. The number of strains and tests performed was increased with each study in an effort to expand the panel of representative strains and susceptibility profiles that could be tested on our system. In addition to evaluating day-to-day reproducibility in each study, we also assessed more general criteria such as categorical agreement with disk diffusion and rate of minor, major, and very major errors. We aimed to achieve the following criteria: ≥95% and ≥90% categorical agreement for the reproducibility and clinical studies, respectively, ≥95% reproducibility, ≤3% major error, ≤2% very major error [13]. A minor error is a discrepancy in which the reference result is susceptible or resistant and our system result is intermediate, or vice versa. A major error is a discrepancy in which the reference result is susceptible, but the system result is resistant. A very major error is a discrepancy in which the reference result is resistant, but the system result is susceptible. Categorical agreement was calculated by dividing the number of agreements between our system and disk diffusion results by the number of tests performed. Reproducibility was calculated by totaling the number of reproducible ProMax results among all tested strains and dividing by the total number of tests performed. For example, if an isolate produced a susceptible result two times and a resistant result one time, the two susceptible results were counted as reproducible results. Isolates were tested against three representative antibiotics of three different classes (fluoroquinolones, aminoglycosides, and carbapenems) commonly prescribed for complicated urinary tract infections and sepsis: ciprofloxacin, gentamicin, and meropenem [14–17]. Each reproducibility study allowed us to optimize the initial algorithm described below to be validated with clinical isolate testing.

#### Clinical validation

The ProMax was evaluated with contemporary (collected within 6 months prior to testing) and stock (no time requirement) strains collected from clinical sites.

### Bacterial strains, media, antibiotics and reagents

**Fig 1:**
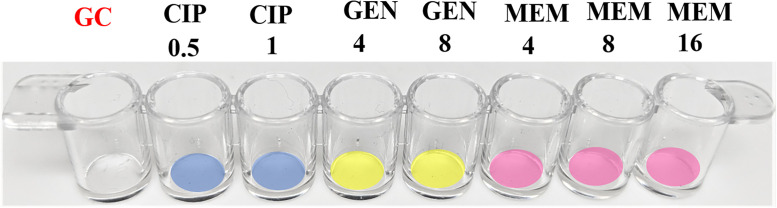
Antibiotic stripwell configuration. Antibiotic concentration listed in μg/mL for each corresponding well number. GC: Growth Control, CIP: Ciprofloxacin, GEN: Gentamicin, MEM: Meropenem.

The clinical isolate panel used in the described studies totaled 161 isolates, including 3 ATCC strains, 39 CDC AR Bank strains, 19 NYPQ strains, 99 MCW strains, and 1 IHMA strain, and consisted of the following organisms: *Citrobacter freundii*, *Enterobacter cloacae*, *Escherichia coli*, *Klebsiella aerogenes*, *Klebsiella oxytoca*, *Klebsiella pneumoniae*, *Morganella morganii*, *Proteus mirabilis*, *Pseudomonas aeruginosa*, and *Serratia marcescens*. Clinical isolates were obtained from de-identified remnant patient samples collected for routine culture. All strains were stored as glycerol stocks at -80°C and were grown from these stocks at 35°C on tryptic soy agar plates with 5% sheep’s blood for 16–24 hours before testing. Samples to be tested on the ProMax system were prepared from second subcultures of frozen stocks. Samples were tested against ciprofloxacin (CIP; Cayman Chemical Company, Ann Arbor, MI, USA), gentamicin (GEN; Sigma-Aldrich, St. Louis, MO, USA), and meropenem (MEM; Cayman Chemical Company). Stripwells were prepared by drying antibiotics in 0.45% saline onto EIA/RIA 8-well strips (Corning, Corning, NY, USA) at the following concentrations as shown in Fig 1: CIP 0.5, 1 μg/mL; GEN 4, 8 μg/mL; MEM 4, 8, 16 μg/mL. The first well of each stripwell was left without antibiotic to be used as a growth control during the assay.

The AST reagent kit contained enough reagents for four runs of the ProMax and consisted of 1M NaOH, 1M HCl, anti-FITC conjugated horseradish peroxidase (HRP; Sigma-Aldrich), cation-adjusted Mueller-Hinton II broth (Teknova, Hollister, CA, USA), and 3,3’,5,5’-tetramethylbenzidine (TMB)-H_2_O_2_ (Neogen, Lexington, KY, USA).

### AST sensor chip

Electrochemical sensor chips with oligonucleotide probes were produced in-house by deposition of gold onto a plastic substrate as previously described [18]. This AST assay was designed to be performed with a gram-negative clinical isolate of known identity; therefore, group-specific sensor chips were used for gram-negative rods. All sensors on these chips were functionalized with specific pairs of *Enterobacterales* and *Pseudomonas aeruginosa* capture and detector probes to cover the ten indicated species [19]. Amperometric detection of 16S rRNA was similar to previously described methods [20,21].

### ProMax AST assay

**Fig 2:**
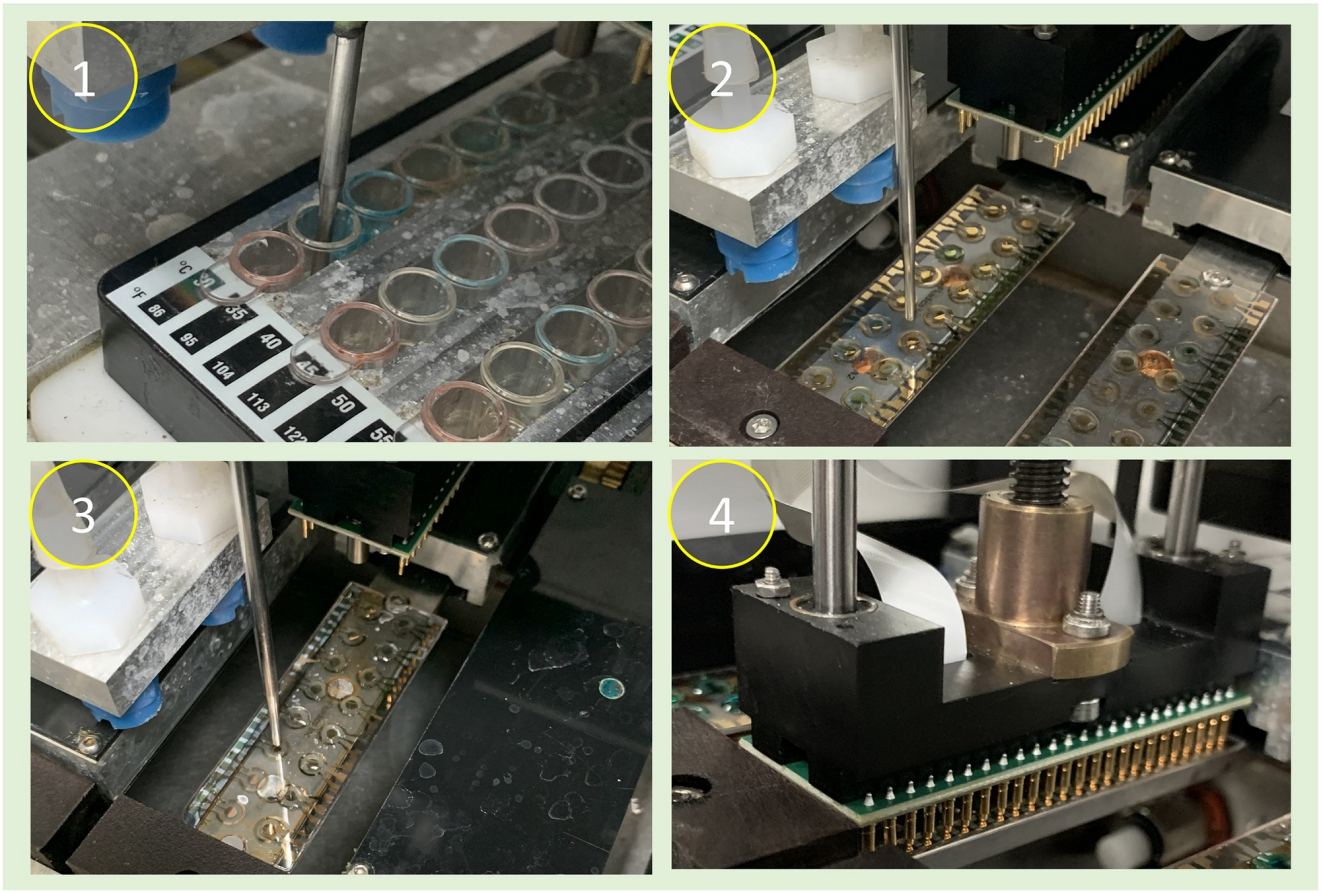
ProMax automated AST assay. (1) Probe delivers inoculum to stripwell after sample dilution step, followed by a 2-hour incubation. (2) After lysing steps, probe delivers lysate to sensor chip. (3) Enzyme and TMB steps performed after hybridization. (4) Electrochemical reading.

Three samples were tested simultaneously in each run of our current system. Isolated colonies from 18–24 hour old plates were directly suspended into 0.45% saline and adjusted to 0.5 McFarland units using a Grant DEN-1B densitometer (Grant Instruments, Cambridge, UK). The growth method, in which organisms are grown in broths prior to testing to reach the exponential phase, was not used in the presented study as this does not reflect the clinical reality that bacteria may not already be in that growth phase at the start of testing. A 1-mL aliquot of each suspension was then placed into the system, along with all testing consumables. Using the graphical user interface (GUI), operators entered sample information and initiated the assay, after which no intervention was required as all the following steps were performed by the system (Fig 2, S2 Fig). The inoculum of each sample was diluted automatically to the species-specific starting concentration by the system, with most species starting at approximately 1 × 10^7^ CFU/mL, rather than the conventional inoculum concentration of 5 × 10^5^ CFU/mL used for microdilution, to account for the short antibiotic exposure time. The system then delivered the inoculum into each well of its corresponding AST stripwell to be incubated for 2 hours at 35°C. The inoculum in each well was lysed after the antibiotic exposure to release the RNA content and the lysate from each well was delivered to its corresponding sensors on two AST sensor chips (a total of 4 sensors per well). Negative and positive control sensors functionalized with capture and detector probes were included on each sensor chip. No sample was delivered to these sensors. The positive control sensor was functionalized with an additional complementary oligonucleotide to generate elevated signal level in a fully functional test. Lysate on all six chips was incubated for 30 minutes at 43°C, during which the RNA hybridized to both the bound capture probe and free detector probe conjugated with fluorescein. After hybridization, a stringency wash was applied to chips to remove non-specific binding. Sensor chips were then dried with a stream of pressurized air. The system then delivered horseradish peroxidase enzyme conjugated to an anti-fluorescein antibody to all sensors, including positive and negative controls. After a second wash and dry cycle to remove unbound enzyme, TMB was delivered to each sensor on every chip. The electrochemical signal amplified by the enzymatic cycling chemical reaction of the HRP with the TMB and the applied voltage across the gold electrodes was measured by the system’s integrated 16-channel potentiostat reader. The readings were used by the built-in algorithm to report a qualitative susceptibility result of “R” (resistant), “I” (intermediate), or “S” (susceptible) as defined by CLSI for each sample against each antibiotic, and results were displayed on the GUI and printout.

### Statistical analysis

Signal generated from each antibiotic condition was analyzed using a built-in reporting algorithm. Before reporting susceptibility, the algorithm first assessed positive, negative, and growth controls, as well as outliers. If either the negative or positive control was out of the acceptable range, the algorithm reported an internal control failure, indicating inability to report the susceptibility. The algorithm then assessed the signal level generated by the growth control (GC) and reported “GC Fail” for any samples with an average GC signal level below a cutoff value. If all controls passed, the average signal from each antibiotic condition was then normalized to the average signal from the growth control, producing a GC ratio for each antibiotic condition. Each antibiotic had two or three GC ratios (one for each concentration/condition) that were compared to predetermined ratio reporting ranges that assign each result to a categorical susceptibility. Specifically, if the GC ratio for a given antibiotic fell below, within, or above the ratio reporting range specific to that antibiotic, the algorithm reported a result of “Susceptible”, “Intermediate”, or “Resistant”, respectively.

To determine the typical microbial response of a susceptible or resistant strain of each species to each antibiotic, we tabulated the GC ratios generated for each antibiotic condition throughout initial correlation and optimization studies. Continued testing through reproducibility studies allowed us to establish the numerical boundaries necessary to distinguish susceptible, intermediate, and resistant strains of each species. Categorical susceptibility is ultimately determined by the established species- and antibiotic-specific ratio reporting ranges set in the built-in algorithm.

## Results

### Susceptibility interpretation using GC ratios

**Fig 3:**
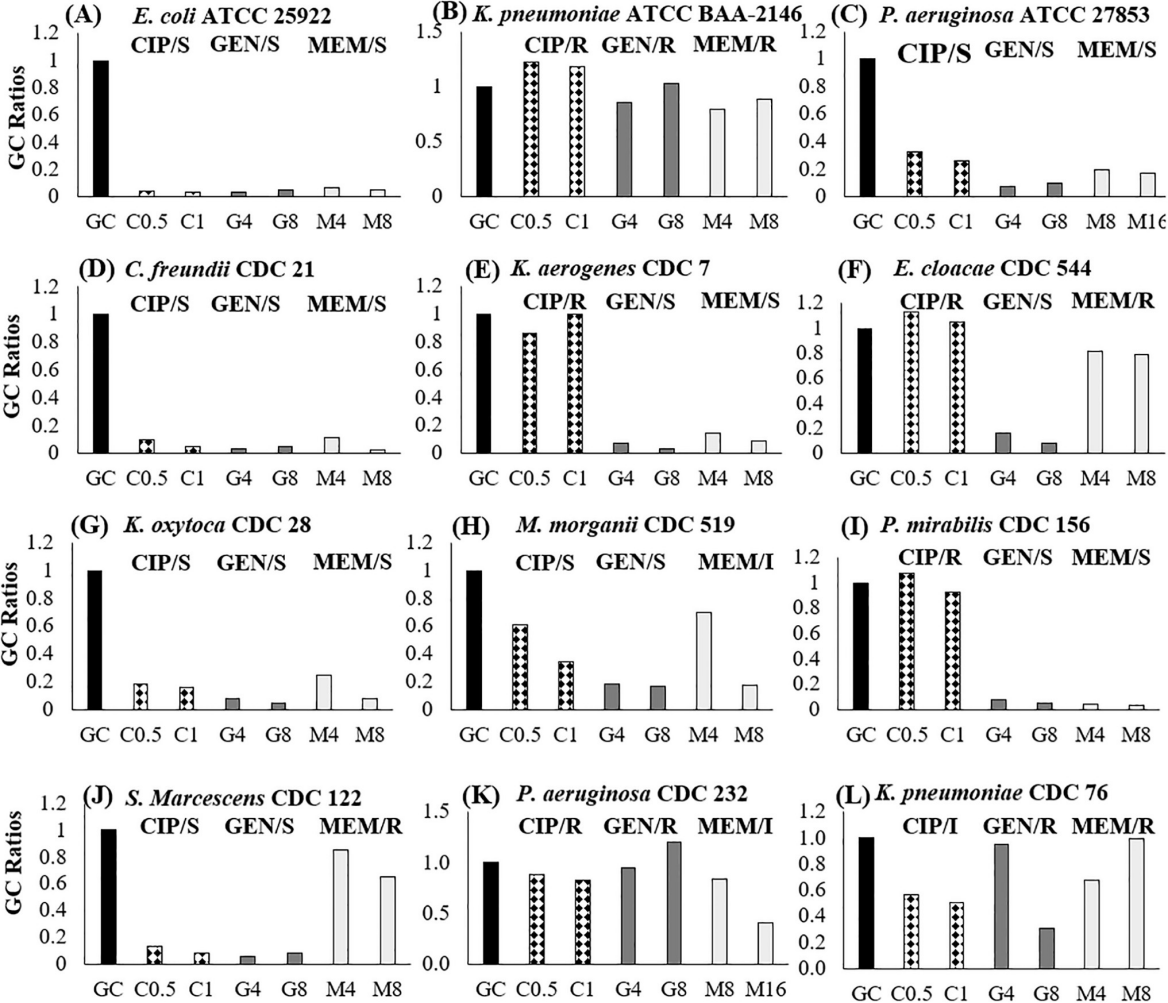
GC ratios for example antibiotic-microbial combinations. GC ratios for each condition–ciprofloxacin (CIP) 0.5 and 1 μg/mL, gentamicin (GEN) 4 and 8 μg/mL, and meropenem (MEM) 4 (tested for *Enterobacterales* only), 8, and 16 (tested for *Pseudomonas aeruginosa* only)–demonstrate susceptible (S), intermediate (I), and resistant (R) responses.

Shown in Fig 3 are examples of various antibiotic-microbial responses observed during testing. GC ratio reporting ranges differed among organism-antibiotic combinations with a majority of species having a range of 0.6 to 0.8 for both ciprofloxacin and meropenem and 0.3 to 0.4 for gentamicin. Values below, within, and above these ranges indicated a susceptible, intermediate, or resistant result, respectively. These quantitative cutoff values were incorporated into the system’s built-in algorithm used for later clinical testing.

### Reproducibility studies

A total of 43 strains (39 CDC, 3 ATCC, 1 IHMA) covering all ten target species were tested across three separate reproducibility studies to report a total 346 susceptibility results (per antibiotic) compared to the disk diffusion reference method. A percentage distribution of susceptibilities for each study is shown below in Table 1. As mentioned above, studies 1, 2, and 3 had a panel of 28, 39, and 39 strains tested 2–3 each times for a total of 62, 116, and 168 results (total 346), respectively, per antibiotic. A summary of the three studies is shown in Table 2. The distribution of species was as shown in S1 Table. Repeated testing and validation of optimization efforts including higher AST inoculum and antibiotic concentrations led to a final overall categorical agreement and reproducibility of 94.0% and 96.4% by the end of Reproducibility Study 3.

**Table 1:** Distribution of susceptibilities in each reproducibility study.

	Susceptible	Intermediate	Resistant
Reproducibility Study 1			
Ciprofloxacin	9 (32.1%)	0 (0.0%)	19 (67.9%)
Gentamicin	16 (57.1%)	1 (3.6%)	11 (39.3%)
Meropenem	11 (39.3%)	0 (0.0%)	17 (60.7%)
Reproducibility Study 2			
Ciprofloxacin	15 (38.5%)	0 (0.0%)	24 (61.5%)
Gentamicin	24 (61.5%)	1 (2.6%)	14 (35.9%)
Meropenem	15 (38.5%)	1 (2.6%)	23 (58.9%)
Reproducibility Study 3			
Ciprofloxacin	15 (38.5%)	0 (0.0%)	24 (61.5%)
Gentamicin	23 (58.9%)	2 (5.1%)	14 (35.9%)
Meropenem	16 (41.0%)	1 (2.6%)	22 (56.4%)

Number of susceptible, intermediate, and resistant isolates tested in each reproducibility study.

**Table 2:** Summary of reproducibility studies.

Antibiotic	Minor Errors	Major Errors	Very Major Errors	Categorical Agreement	Reproducibility
Reproducibility Study One					
Ciprofloxacin	1	0	4	57/62 (91.9%)	60/62 (96.8%)
Gentamicin	4	1	2	55/62 (88.7%)	60/62 (96.8%)
Meropenem	1	4	6	51/62 (82.3%)	56/62 (90.3%)
TOTAL	6	5	12	163/186 (87.6%)	176/186 (94.6%)
Reproducibility Study Two					
Ciprofloxacin	3	1	7	105/116 (90.5%)	115/116 (99.1%)
Gentamicin	5	3	1	107/116 (92.2%)	111/116 (95.7%)
Meropenem	5	3	11	97/116 (83.6%)	106/116 (91.4%)
TOTAL	13	7	19	309/348 (88.8%)	332/348 (95.4%)
Reproducibility Study Three					
Ciprofloxacin	2	2	2	162/168 (96.4%)	163/168 (97.0%)
Gentamicin	3	3	0	162/168 (96.4%)	164/168 (97.6%)
Meropenem	7	7	4	150/168 (89.3%)	159/168 (94.6%)
TOTAL	12	12	6	474/504 (94.0%)	486/504 (96.4%)

Categorical agreement, reproducibility, and discrepancy summary for each antibiotic in each study.

Although the categorical agreements for ciprofloxacin and gentamicin were >95% and the very major and major error rates for all three antibiotics were <5%, meeting the criteria, meropenem only had an 89.3% categorical agreement. Most discrepancies were due to low growth control signals produced by isolates with a longer doubling time. Most slow-growing clinical strains can be routinely identified by overnight subculture followed by conventional biochemical reactions or 16S rRNA gene sequence analysis in a diagnostic laboratory setting [22]. Despite the effort to compensate for organisms with longer doubling times by increasing the inoculum concentrations, we decided not to include the meropenem susceptibility for *P*. *mirabilis*, *M*. *morganii*, and *S*. *marcescens* on the AST panel for this initial validation study due to the inconsistent reproducibility to distinguish microbiological responses among those three species without increasing the antibiotic exposure time. The antibiotic-specific categorical agreement and reproducibility from the third study excluding these three problematic species were 100% and 100% for ciprofloxacin, 98.7% and 100% for gentamicin, and 98.5% and 98.5% for meropenem, respectively; the total categorical agreement was 99.1%. Tables 3–5 are breakdown of species-specific discrepancies in the reproducibility studies. In the first reproducibility study, 7 out of 12 (58%) very major errors were reported for *K*. *pneumoniae* as shown in Table 3, but this number was reduced to 8/19 (42%) in the second reproducibility study (Table 4) and 1/6 (17%) in the third reproducibility study (Table 5) while the overall very major error rate was also reduced from 6.5% (12/186) to 5.5% (19/348) and then to 1.2% (6/504).

**Table 3:** Reproducibility study 1 discrepancy summary.

	CIP			GEN			MEM		
Organism	minor	major	very major	minor	major	very major	minor	major	very major
*C*. *freundii*	0	0	0	2	0	0	0	0	0
*E*. *cloacae*	0	0	2	0	0	0	0	0	0
*E*. *coli*	0	0	0	0	0	0	0	0	2
*K*. *aerogenes*	0	0	0	0	0	0	0	0	0
*K*. *oxytoca*	0	0	0	0	0	0	0	0	0
*K*. *pneumoniae*	0	0	2	1	1	2	0	0	3
*M*. *morganella*	0	0	0	0	0	0	0	0	0
*P*. *mirabilis*	1	0	0	0	0	0	0	1	1
*P*. *aeruginosa*	0	0	0	0	0	0	1	1	0
*S*. *marcescens*	0	0	0	1	0	0	0	2	0
Total	1	0	4	4	1	2	1	4	6

Number of minor, major, and very major errors in reproducibility study 1 is listed for all 10 species and 3 antibiotics: ciprofloxacin (CIP), gentamicin (GEN), meropenem (MEM).

**Table 4:** Reproducibility study 2 discrepancy summary.

	CIP			GEN			MEM		
Organism	minor	major	very major	minor	major	very major	minor	major	very major
*C*. *freundii*	0	0	0	2	0	0	0	1	0
*E*. *cloacae*	0	0	4	0	0	0	1	0	0
*E*. *coli*	0	0	0	0	0	1	1	0	2
*K*. *aerogenes*	1	0	0	0	1	0	0	0	0
*K*. *oxytoca*	0	0	0	0	0	0	0	0	0
*K*. *pneumoniae*	1	0	3	2	0	0	3	0	5
*M*. *morganella*	1	0	0	0	0	0	0	0	0
*P*. *mirabilis*	0	0	0	0	0	0	0	0	0
*P*. *aeruginosa*	0	1	0	0	2	0	0	1	0
*S*. *marcescens*	0	0	0	1	0	0	0	1	4
Total	3	1	7	5	3	1	5	3	11

Number of minor, major, and very major errors in reproducibility study 2 is listed for all 10 species and 3 antibiotics: ciprofloxacin (CIP), gentamicin (GEN), meropenem (MEM).

**Table 5:** Reproducibility study 3 discrepancy summary.

	CIP			GEN			MEM		
Organism	minor	major	very major	minor	major	very major	minor	major	very major
*C*. *freundii*	1	1	0	0	1	0	0	2	0
*E*. *cloacae*	0	0	2	0	0	0	0	0	0
*E*. *coli*	0	0	0	2	2	0	2	1	1
*K*. *aerogenes*	0	0	0	0	0	0	0	0	0
*K*. *oxytoca*	0	0	0	0	0	0	0	0	0
*K*. *pneumoniae*	0	0	0	0	0	0	4	0	1
*M*. *morganella*	1	0	0	0	0	0	0	0	0
*P*. *mirabilis*	0	0	0	1	0	0	0	1	2
*P*. *aeruginosa*	0	1	0	0	0	0	0	2	0
*S*. *marcescens*	0	0	0	0	0	0	1	1	0
Total	2	2	2	3	3	0	7	7	4

Number of minor, major, and very major errors in reproducibility study 3 is listed for all 10 species and 3 antibiotics: ciprofloxacin (CIP), gentamicin (GEN), meropenem (MEM).

### Clinical validation

Since we do not intend to report the meropenem susceptibility for *P*. *mirabilis*, *M*. *morganii*, and *S*. *marcescens* with the current rapid AST test with a 2-hour antibiotic exposure time, we did not further optimize the algorithm necessary to determine categorical agreement for these species against meropenem. However, we did test and collect meropenem susceptibility raw data from ProMax during the clinical validation study for future analysis. The categorical agreement between ProMax and disk diffusion results for ciprofloxacin, gentamicin, and meropenem was 94.9%, 99.2%, and 86.4%, respectively, including meropenem susceptibility for *P*. *mirabilis*, *M*. *morganii*, and *S*. *marcescens* as shown in Table 6. The categorical agreement for meropenem was 94.0% if excluding *P*. *mirabilis*, *M*. *morganii*, and *S*. *marcescens*. Additionally, we calculated the rate of major and very major errors for each antibiotic. Ciprofloxacin and gentamicin results both show major and very major error rates of 0% as shown in Table 7. However, meropenem produced a major error rate of 11.9% (12 out of 101 susceptible isolates) and a very major error rate of 26.7% (4 out of 15 resistant isolates). Even after excluding meropenem susceptibility for *P*. *mirabilis*, *M*. *morganii*, and *S*. *marcescens*, the major and very major error rates were 2.9% (2 out of 68 susceptible isolates) and 21.4% (3 out of 14 resistant isolates), respectively, indicating the limitation of rapid AST with time-dependent antimicrobials such as carbapenem class antibiotics.

**Table 6:** Results from clinical validation using NYPQ and MCW isolates.

Organism	Number of isolates	Disk Diffusion Frequency									CA (%)		
		CIP			GEN			MEM			CIP	GEN	MEM
		S	I	R	S	I	R	S	I	R			
*C*. *freundii*	13	6	1	6	8	0	5	11	0	2	100	100	92.3
*E*. *cloacae*	11	4	0	7	9	0	2	7	1	3	100	100	81.8
*E*. *coli*	14	6	2	6	8	0	6	13	0	1	85.7	100	92.9
*K*. *aerogenes*	6	6	0	0	6	0	0	4	0	2	83.3	100	100
*K*. *oxytoca*	10	5	0	5	6	0	4	10	0	0	100	100	100
*K*. *pneumoniae*	14	5	1	8	14	0	0	11	1	2	100	92.9	100
*M*. *morganii*	14	6	2	6	11	0	3	14	0	0	85.7	100	100
*P*. *aeruginosa*	16	7	1	8	13	0	3	12	0	4	93.8	100	93.8
*P*. *mirabilis*	9	1	0	8	5	0	4	9	0	0	100	100	88.9
*S*. *marcescens*	11	6	0	5	11	0	0	10	0	1	100	100	9.1
Total	118	52	7	59	91	0	27	101	2	15	94.9	99.2	86.4

Disk diffusion results and categorical agreement (CA) shown for each species and antibiotic combination.

**Table 7:** Summary of discrepancies for clinical testing.

	CIP			GEN			MEM		
Organism	Minor	major	very major	minor	major	very major	minor	major	very major
*C*. *freundii*	0	0	0	0	0	0	0	0	1
*K*. *aerogenes*	1	0	0	0	0	0	0	0	0
*E*. *cloacae*	0	0	0	0	0	0	0	0	2
*E*. *coli*	2	0	0	0	0	0	0	1	0
*K*. *oxytoca*	0	0	0	0	0	0	0	0	0
*K*. *pneumoniae*	0	0	0	1	0	0	0	0	0
*M*. *morganii*	2	0	0	0	0	0	0	0	0
*P*. *aeruginosa*	1	0	0	0	0	0	0	1	0
*P*. *mirabilis*	0	0	0	0	0	0	0	1	0
*S*. *marcescens*	0	0	0	0	0	0	0	9	1
Total Number	5	0	0	1	0	0	0	12	4
Percentage of 118 isolates	4.2	0.0	0.0	0.8	0.0	0.0	0.0	10.2	3.4
Percentage of R isolates	N/A	N/A	0.0 (0/59)	N/A	N/A	0.0 (0/27)	N/A	N/A	26.7 (4/15)
Percentage of S isolates	N/A	0.0 (0/52)	N/A	N/A	0.0 (0/91)	N/A	N/A	11.9 (12/101)	N/A

Number of minor, major, and very major errors in clinical testing is listed for all 10 species and 3 antibiotics: ciprofloxacin (CIP), gentamicin (GEN), meropenem (MEM). Rate of each discrepancy type is calculated in the bottom section.

## Discussion

Changes in RNA in response to antibiotic exposure have been shown to be both significant and rapid, but most molecular analysis approaches have not been able to demonstrate the distinct microbial responses among clinical strains with MIC values on or near resistant and susceptible breakpoints [23,24]. Additionally, certain UTI pathogens may have slower growth rates and smaller microbiological differences in growth control and treated conditions [25,26]. These bacteria are difficult to detect without examination of the urine using Gram stain, an important lab procedure in microbiology used to differentiate between gram-positive and negative organisms, since many FDA-cleared AST panels include either only gram-positive or negative organisms [27,28]. Therefore, the main goal of this initial evaluation study was to assess the ability to demonstrate substantial categorical agreement between our system and the CLSI disk diffusion reference method on gram-negative clinical strains with MIC values on or near resistant and susceptible breakpoints.

We acknowledged that our short two-hour antibiotic exposure time would necessitate the adjustment of assay parameters, starting with the antibiotic-microbial ratio, to accelerate bacterial response dynamics. Prior to this study, we encountered difficulty in distinguishing between meropenem-resistant and -susceptible strains when testing with stripwell concentrations of 1, 2, and 4 μg/mL meropenem. Drugs that exhibit time-dependent activity (β-lactams and vancomycin) have relatively slow bactericidal action even with the CLSI-recommended 16–24 hours of antibiotic exposure time and the goal of AST is to predict the clinical success or failure of the antibiotic being tested against a particular organism. Therefore, a much-shortened antibiotic exposure time could limit the ability to test all pathogens with clinically relevant susceptibility profiles. We increased the concentration of meropenem fourfold at the start of this validation period and performed the antibiotic exposure at higher inoculum concentrations to collect more microbiological responses at these conditions during the reproducibility studies to determine the direction of parameter adjustments to achieve maximum categorical agreements. We also adjusted the dilution factor to increase the starting concentration to 1x10^7^ CFU/mL for most species and 3x10^7^ CFU/mL for organisms with longer doubling times, such as *P*. *mirabilis*, *M*. *morganii*, and *S*. *marcescens*. While increasing inoculum and antibiotic concentrations showed improvement in agreement and reproducibility, we still observed an especially low agreement and reproducibility for meropenem in each study, never exceeding 90% and 95%, respectively, suggesting the dynamics of microbial response to meropenem are not as rapid as with other antibiotics. Meropenem disrupts cell wall synthesis so its effects may only be measurable when a strain is actively growing, whereas ciprofloxacin and gentamicin act directly on a cell’s nucleic acids and do not require multiple instances of doubling to have an observable effect, which cannot be easily simulated in a rapid AST with a short antibiotic exposure time [29]. Furthermore, we observed frequent discrepancies between our system and disk diffusion when testing *S*. *marcescens* against meropenem. Distinguishing susceptible from resistant isolates of this species proved to be difficult with only a 2-hour AST incubation. Although it may be necessary to extend the incubation time to accommodate these more challenging antibiotic-microbial combinations, a majority of species seemed to have demonstrated successful AST reporting with a 2-hour exposure time, suggesting that there may be other options to investigate in order to include problematic species while keeping this parameter.

Additionally, we modified the species-specific GC ratio reporting ranges in our algorithm to address the failed cases observed throughout each study. Raw data from the reproducibility studies confirmed the difference in growth rates of bacterial species and reaction times of antibiotics. However, rather than extend our AST incubation and total assay time, we modified the GC ratio reporting ranges for each antibiotic and species combination to reflect the changed antibiotic-microbial ratios, which led to an average categorical agreement and reproducibility among all three antibiotics of 99.1% and 99.5% if excluding meropenem susceptibility reporting for *P*. *mirabilis*, *M*. *morganii*, and *S*. *marcescens* respectively, in the final reproducibility study.

With an optimized algorithm and testing conditions, we achieved 94.9%, 99.2%, and 86.4%, (Table 6) categorical agreement for ciprofloxacin, gentamicin, and meropenem, and 94.9%, 99.2%, and 94.0% if excluding meropenem susceptibility reporting for *P*. *mirabilis*, *M*. *morganii*, and *S*. *marcescens* respectively, during clinical validation. However, we observed frequent disagreement between disk diffusion and microdilution performed by MCW using the Vitek 2 system, which calls attention to the potential flaws in the CLSI definitions of “Resistant”, “Intermediate”, and “Susceptible”, meaning that any qualitative AST system such as the ProMax that is not completely identified as either accelerated microdilution or accelerated disk diffusion cannot be easily tied to one reference method. Disagreement between disk diffusion and our system for most isolates (excluding *S*. *marcescens*) may occur due to a wide range of growth rate minimizing the difference in microbiological responses, and therefore, GC ratios, between resistant and susceptible strains. This obstacle may be addressed through continuous collection and testing of clinical isolates, followed by further adjustments to the antibiotic-microbial ratio and reporting algorithm.

Current limitations of our system include a panel of only gram-negative species (three of which require additional investigation to be included in the final panel), an AST panel of only three antibiotics, and a relatively low throughput. Gram-positive organisms require different lysis reagents and a different lysing procedure, which are not within the scope of this study focusing on gram-negative rods. The current system can accommodate up to three samples per run and one AST stripwell containing three antibiotics per sample. This configuration limits the number of antibiotics to be tested and the throughput of the system due to the need for users to replenish consumables after each AST run with a fixed number of sensor chip and stripwell slots. Additionally, this study only tested monomicrobial samples. Polymicrobial samples may be tested using the ID sensor chip, in which each sensor is functionalized with a specific probe pair to detect a certain species on our panel, rather than the AST sensor chip described in this study. Such samples may also still be tested the with described AST sensor chip, since the goal of AST is to determine whether a given antibiotic is appropriate for therapy, not to identify the species within a sample. There would need to be at least one species from our panel present in the polymicrobial sample to obtain a susceptibility result from our system and therefore determine whether a given antibiotic is effective.

The studies presented demonstrate the analytical and clinical feasibility of measuring the phenotypic response of bacteria to antibiotics using a biosensor array chip in an automated system. Although the presented system is only able to perform AST from clinical isolates grown on solid media at a fixed starting inoculum concentration, successful optimization of the current rapid AST assay from clinical isolates suggests that these basic assay principles may potentially be used in future studies of advanced automated assays such as streamlined ID/AST directly from specimen (in which users would load a tube of whole blood or urine obtained during routine collection), AST directly from clinical specimens, heteroresistance detection, time-kill assays, and the testing of polymicrobial samples and multidrug-resistant organisms. Such assays would require further modifications to our system not only to address the limitations described above but also to accommodate different assay objectives and specimen types, which may require one or more centrifugation steps to remove matrix interference components within the specimen, including but not limited to existing antimicrobials in the patient sample.

Our initial evaluation of the current AST assay shows promising potential for the development of a fully automated rapid ID/AST platform direct from clinical specimens and would eliminate the limiting and time-consuming restraints observed in current commercial and conventional methods.

## Supporting information

S1 Fig Total turnaround time (TAT) for ID and AST in clinical microbiology laboratories. (TIF)

S2 Fig ProMax AST system. (TIF)

S1 Table Number of species in reproducibility studies. Number of isolates for each species in each study, including the number of isolates with MIC on the intermediate (I) breakpoint for ciprofloxacin (CIP), gentamicin (GEN), and meropenem (MEM) as confirmed with disk diffusion.
(PDF)
