# Supplementary figures and images for: Feasibility and potential significance of rapid in vitro qualitative phenotypic antimicrobial susceptibility testing of gram-negative bacilli with the ProMax system
Source: PLoS One. 2021 Mar 26;16(3):e0249203. doi: 10.1371/journal.pone.0249203 (PMC7996979; doi:10.1371/journal.pone.0249203)

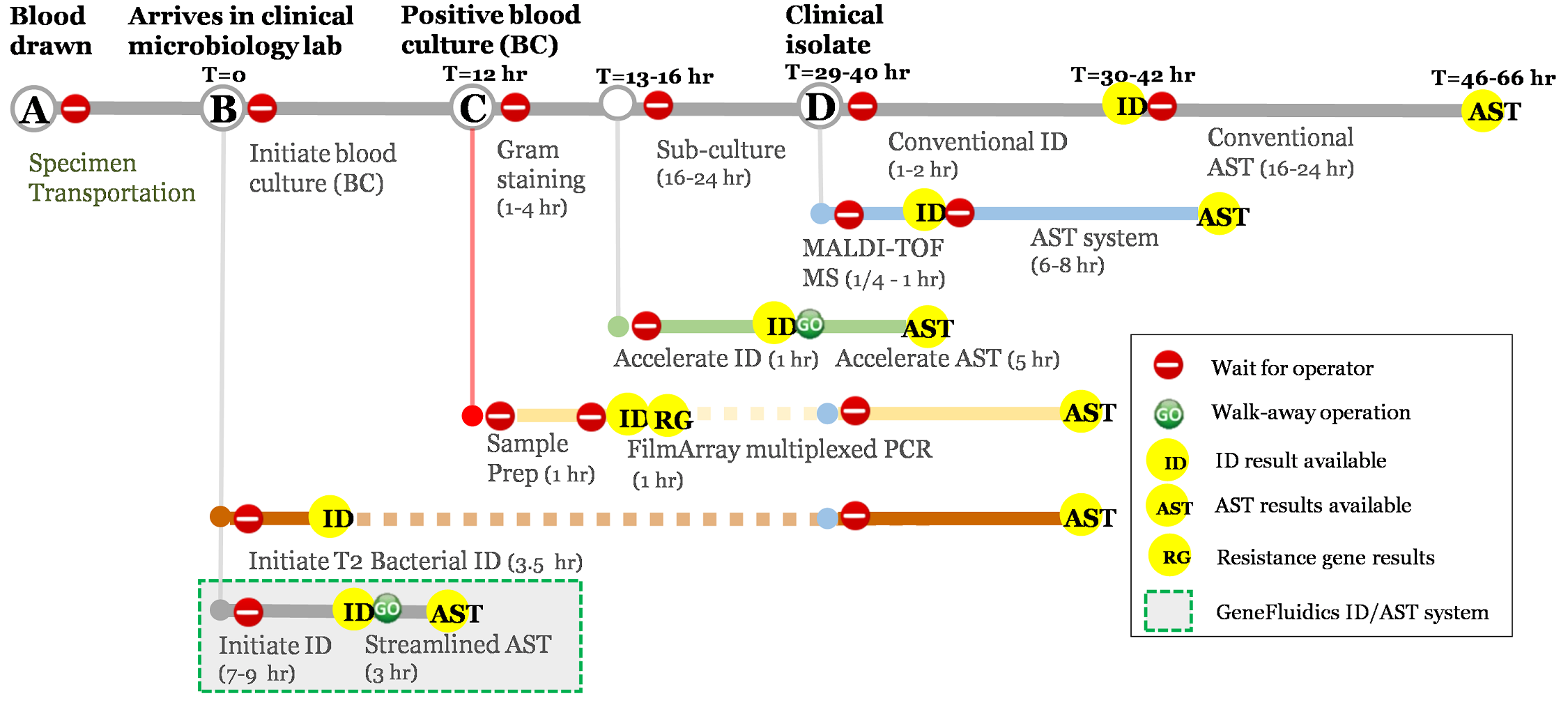

Supplement: S1 Fig — (TIF) [file pone.0249203.s001.tif]

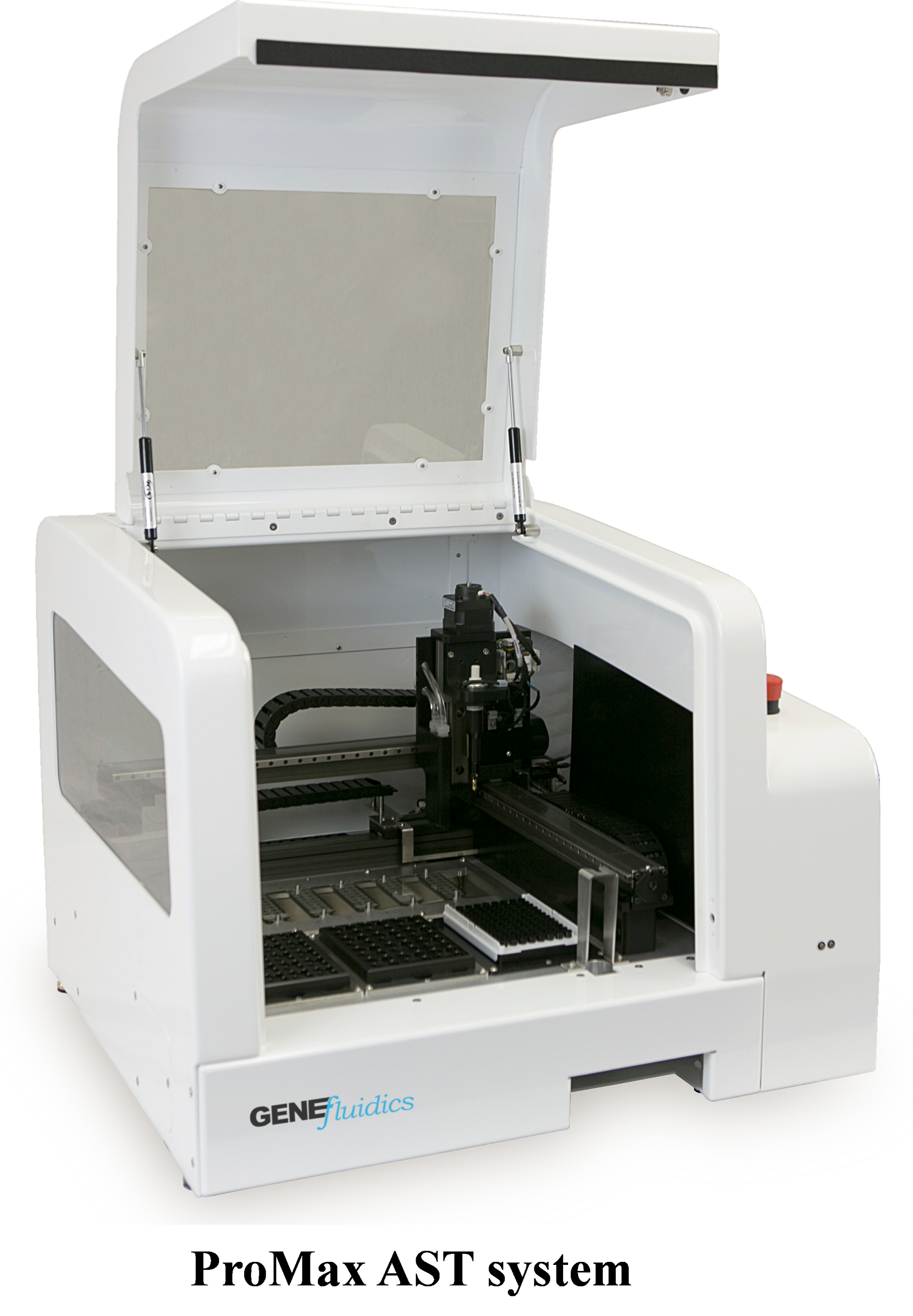

Supplement: S2 Fig — (TIF) [file pone.0249203.s002.tif]
