# Supplementary material for: Feasibility and potential significance of rapid in vitro qualitative phenotypic antimicrobial susceptibility testing of gram-negative bacilli with the ProMax system
Source: PLoS One. 2021 Mar 26;16(3):e0249203. doi: 10.1371/journal.pone.0249203 (PMC7996979; doi:10.1371/journal.pone.0249203)
Supplement: S1 Table — Number of isolates for each species in each study, including the number of isolates with MIC on the intermediate (I) breakpoint for ciprofloxacin (CIP), gentamicin (GEN), and meropenem (MEM) as confirmed with disk diffusion. (PDF) [file pone.0249203.s003.pdf]

S1 Table. Number of species in reproducibility studies.

Organism	Reproducibility Study 1	Reproducibility Study 2	Reproducibility Study 3
C. freundii	1	2	3
E. cloacae	3	5	5
E. coli	4	6	6
K. aerogenes	2	2	2
K. oxytoca	0	1	1
K. pneumoniae	9	12	12
M. morganii	1	1	1
P. aeruginosa	2	3	3
P. mirabilis	2	3	2
S. marcescens	4	4	4
Total	28	39	39
Number of CIP-I isolates	0	0	0
Number of GEN-I isolates	1	1	2
Number of MEM-I isolates	0	1	1

Number of isolates for each species in each study, including the number of isolates with MIC on the intermediate (I) breakpoint for ciprofloxacin (CIP), gentamicin (GEN), and meropenem (MEM) as confirmed with disk diffusion.
